# Clinical Significance of an Incidentally Detected Lateral Lingual Foramen on Cone-Beam Computed Tomography (CBCT): A Case Report and Review of Pertinent Literature

**DOI:** 10.7759/cureus.99725

**Published:** 2025-12-20

**Authors:** Sundar Ramalingam, Abdulrahman A Alamri, Ali A Almujali, Abdullah Alqarni, Abdallah G Alalwan, Faisal M Alrefaei

**Affiliations:** 1 Oral and Maxillofacial Surgery, College of Dentistry, King Saud University, Riyadh, SAU; 2 Oral and Maxillofacial Surgery, Dental University Hospital, King Saud University Medical City, Riyadh, SAU; 3 Oral and Maxillofacial Surgery, Al-Iman General Hospital, Ministry of Health (Riyadh First Health Cluster), Riyadh, SAU; 4 Oral and Maxillofacial Surgery, Prince Sultan Military Medical City, Riyadh, SAU; 5 Oral and Maxillofacial Surgery, College of Dentistry, King Khalid University, Abha, SAU; 6 Oral and Maxillofacial Surgery, Yonsei University Dental Hospital, Seoul, KOR

**Keywords:** cbct, dental implantology, lateral lingual canal, lateral lingual foramen, mandible, oral surgery

## Abstract

The lateral lingual foramen (LLF) is an often-overlooked anatomical variant of the mandible with important clinical implications during implant placement and oral surgical procedures. Unlike the consistently present midline lingual foramen, LLF exhibits marked variability in its prevalence, morphology, and neurovascular content, contributing to potential complications when unrecognized. This case report describes an incidental LLF identified during routine cone-beam computed tomography (CBCT) undertaken for the preoperative evaluation of mandibular third molars in a healthy 21-year-old woman. CBCT revealed a distinct LLF (0.93 mm in diameter) positioned lingual to the mental foramen and apical to the premolar roots, with a corresponding lateral lingual canal (LLC), 6.39 mm in length. The canal demonstrated continuity between the lingual cortical bone and soft tissues of the floor of the mouth, suggesting the presence of a neurovascular bundle branching from the mental or inferior alveolar neurovascular complex. The patient subsequently underwent uneventful maxillary extractions and mandibular coronectomies, after which she was advised regarding the significance of the incidental LLF for any future osteotomy, genioplasty, or implant procedures in the interforaminal region. A review of pertinent literature highlights wide variability in LLF prevalence, typical localization in the canine-premolar region, and the potential for significant hemorrhage, especially when the LLF diameter exceeds 1 mm or when associated with atrophic mandibles. CBCT consistently emerges as the imaging modality of choice for accurate detection, morphological characterization, and preoperative risk assessment. Recognition of LLF and LLC is critical for preventing bleeding complications, neurosensory deficits, and life-threatening airway compromise during anterior mandibular surgery and implantology.

## Introduction

The mandible, a critical structure in craniofacial anatomy, harbors numerous foramina that serve as conduits for neurovascular bundles [1]. Among these, the lingual foramen has been extensively studied due to its clinical relevance in dental implantology and oral surgery [2]. However, the lateral lingual foramen (LLF), a less frequently discussed anatomical feature, has recently garnered attention for its potential role in surgical complications and hemodynamic disturbances [3]. Unlike the midline lingual foramen, which is consistently present, the LLF exhibits considerable variability in its prevalence, location, and morphology, making it a subject of both anatomical curiosity and clinical concern [4].

The mandibular lingual cortex is perforated by multiple foramina, including the median and lateral lingual foramina, which transmit branches of the sublingual and submental arteries, as well as sensory nerve fibers [4,5]. Among them, the LLF is defined as an accessory foramen located on the lingual cortical surface of the mandible, lateral to the midline, and is frequently associated with a bony conduit, the lateral lingual canal (LLC) [6]. The LLC may transmit branches of the submental or sublingual arteries, veins, and occasionally sensory nerve fibers. In contrast, the median lingual foramen (MLF) is a consistently present midline structure located near the genial tubercles and is typically associated with the median lingual canal (MLC) [3]. While the MLF is well-documented, the LLF remains understudied, with conflicting reports on its incidence and anatomical characteristics [7,8]. Some studies suggest that the LLF is present in a significant proportion of individuals, often located in the canine-premolar region, along the lingual cortical plate [4,9-11]. Others report its absence or inconsistent appearance, raising questions about its embryological development and functional significance [12].

A critical gap in the literature lies in the lack of standardized criteria for identifying and classifying the LLF across imaging modalities. Conventional radiography often fails to detect smaller or obliquely oriented foramina, whereas cone-beam computed tomography (CBCT) provides higher resolution and three-dimensional visualization, enabling more accurate assessments [2,3,9,13,14]. CBCT voxel resolution and measurement reliability directly influence the detection of small or obliquely oriented lingual foramina; canals with a diameter greater than 1.0 mm are commonly regarded as higher risk for hemorrhage and therefore warrant modified surgical planning [15]. Despite these advancements, discrepancies persist regarding the optimal imaging parameters and diagnostic thresholds for distinguishing the LLF from other mandibular foramina [11]. Furthermore, the clinical implications of the LLF, particularly its role in hemorrhage risk during implant placement or osteotomy procedures, remain inadequately explored, with few studies correlating anatomical findings with surgical outcomes [4,7]. From a clinical perspective, the LLF assumes particular significance during surgical procedures involving the anterior and premolar regions of the mandible [13]. These include dental implant placement in the interforaminal region, genioplasty, anterior mandibular osteotomies, flap elevation on the lingual cortex, and procedures performed in atrophic or edentulous mandibles [16]. Unrecognized injury to the neurovascular contents of the LLF or LLC may result in rapid floor-of-mouth hemorrhage, hematoma formation, airway compromise, or neurosensory disturbances [1,13,16]. Consequently, awareness of this anatomical variation is essential for safe surgical planning and risk mitigation.

The objective of this case report is to present an incidental radiographic finding of a LLF and LLC in a healthy young adult woman referred for elective orthodontic extractions. This case is presented to increase clinical awareness of an often-overlooked mandibular anatomical variation that may have important implications during oral surgery and implant placement. In addition, this article reviews relevant literature to support the recommendation that clinicians routinely evaluate for their presence on preoperative imaging, ultimately guiding safer and more predictable surgical interventions. The significance of this work extends to both clinicians and researchers, offering insights that may reduce procedural risks and inform future investigations into mandibular neurovascular anatomy.

## Case presentation

A 21-year-old woman was referred to the oral and maxillofacial surgery outpatient clinic for planned surgical removal of all third molars prior to orthodontic treatment. The patient had no relevant medical history and reported no systemic illnesses, familial disorders, or parafunctional habits.

**Figure 1:**
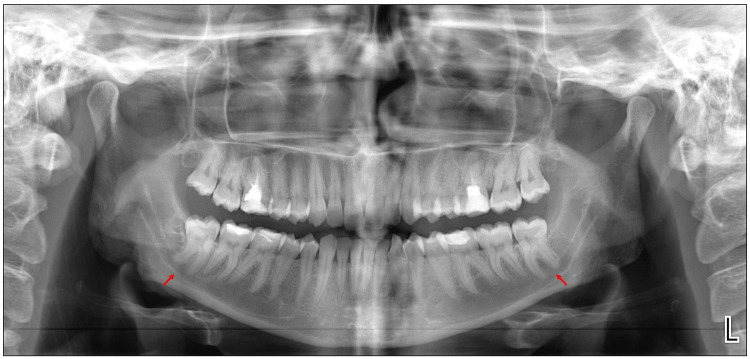
Dental panoramic radiograph (OPG). Preoperative orthopantomogram (OPG) showing bilateral mandibular third molars (red arrows) in close proximity to the inferior alveolar nerve (IAN) canal. No lateral lingual foramen (LLF) or lateral lingual canal (LLC) is visible on this two-dimensional imaging modality.

Clinical examination revealed buccoverted maxillary third molars and partially erupted mandibular third molars. A panoramic radiograph demonstrated close proximity of the mandibular third molar roots to the inferior alveolar nerve (IAN) canal bilaterally (Figure 1). To further assess the relationship between the third molars and the IAN canal, a CBCT scan of the mandible was obtained. The CBCT scan was acquired using a Planmeca ProMax® 3D unit (Planmeca Oy, Helsinki, Finland) with a large field of view (up to 20 × 20 cm) encompassing the mandible bilaterally from ramus to ramus, extending superiorly from the occlusal plane to the inferior border of the mandible. High-resolution imaging was performed with a voxel size ranging from 0.2 to 0.3 mm. All multiplanar reconstructions and linear measurements were obtained using Planmeca Romexis Dental Imaging Software (Planmeca Oy, Helsinki, Finland).

**Figure 2:**
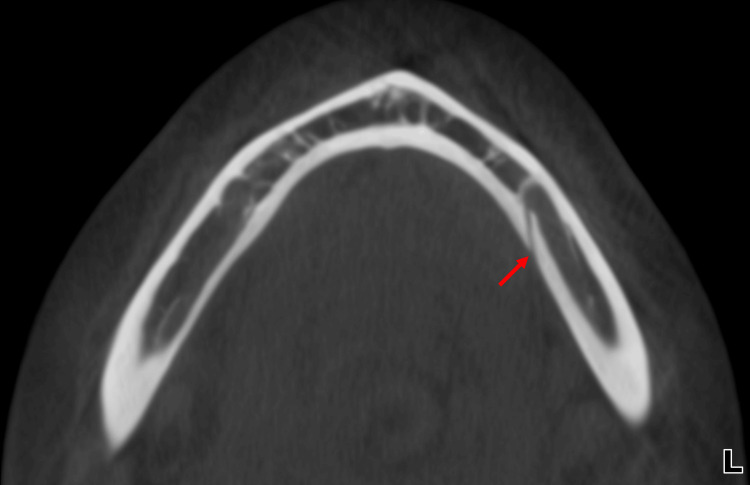
Cone-beam computed tomography (CBCT) imaging of the mandible. Axial section showing lateral lingual foramen (LLF) and lateral lingual canal (LLC) in the left mandible (red arrow) and also the intrabony trajectory of the LLC along the lingual cortical plate.

**Figure 3:**
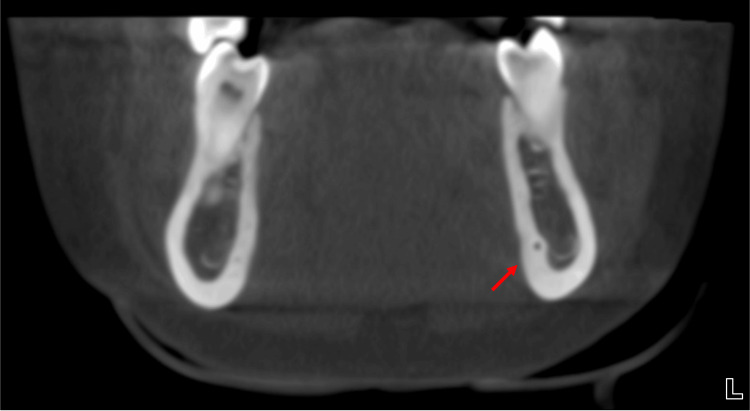
Cone-beam computed tomography (CBCT) imaging of the mandible. Coronal section through the bilateral premolar areas showing lateral lingual foramen (LLF) and lateral lingual canal (LLC) in the left mandible (red arrow), along with a well-distinguishable LLC on the lingual cortex only in the left premolar area.

**Figure 4:**
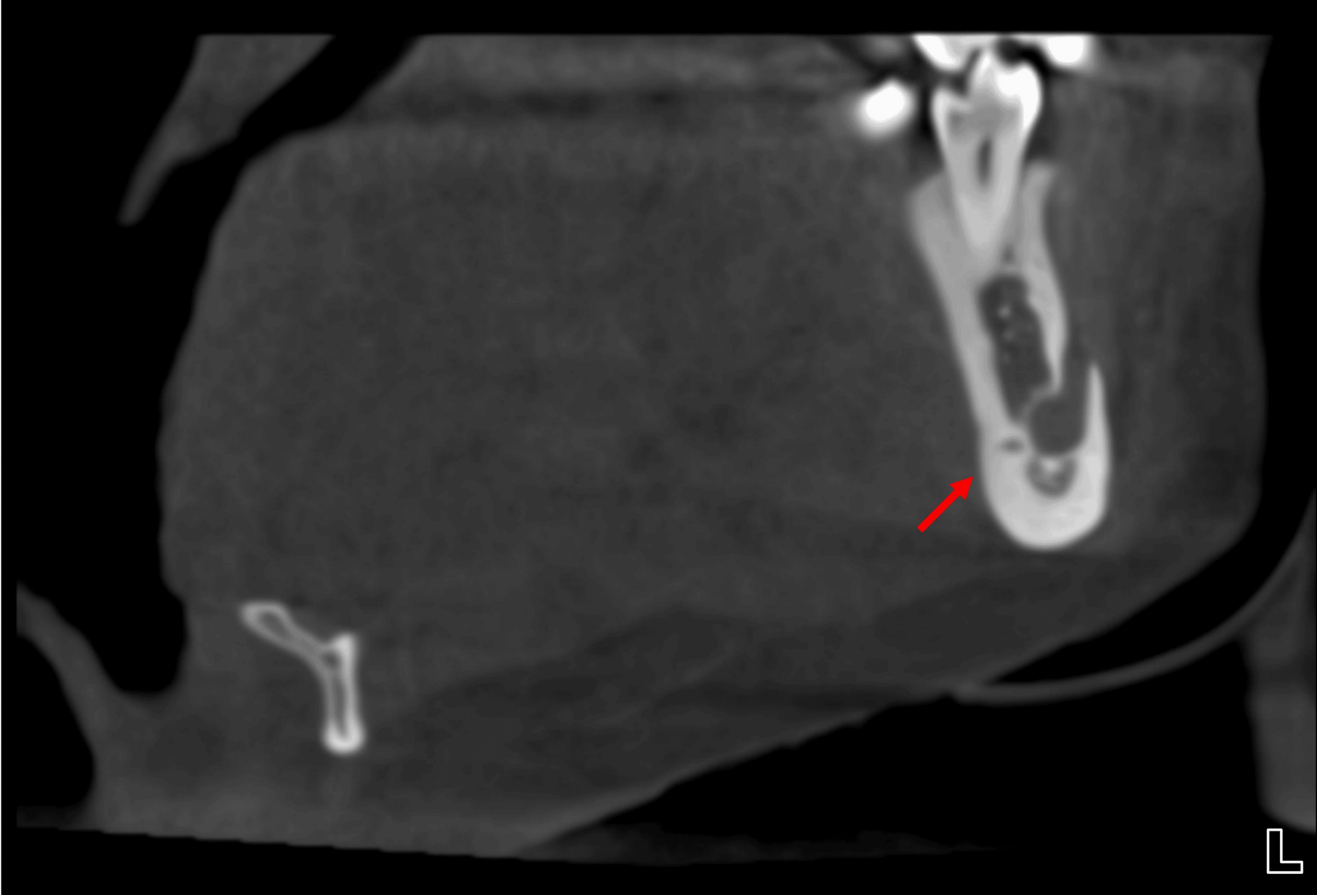
Cone-beam computed tomography (CBCT) imaging of the mandible. Coronal section through the left mental foramen and inferior alveolar nerve (IAN) canal showing lateral lingual foramen (LLF) and lateral lingual canal (LLC) in the left mandible (red arrow) and highlighting the proximity of the LLC to adjacent neurovascular structures.

**Figure 5:**
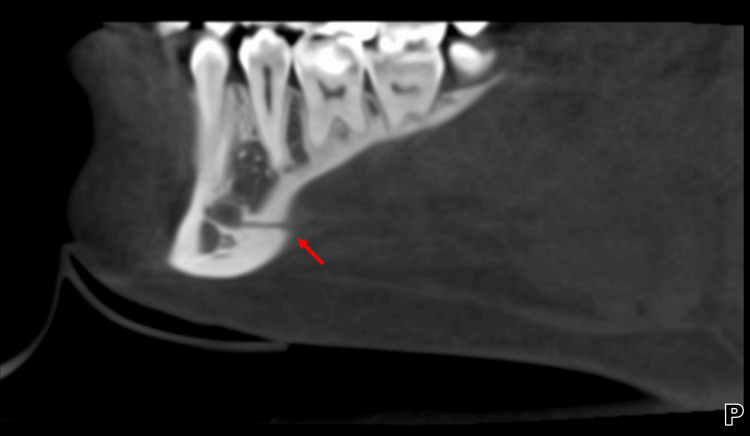
Cone-beam computed tomography (CBCT) imaging of the mandible. Sagittal section of the left mandible showing the lateral lingual foramen (LLF) and lateral lingual canal (LLC) in the left mandible (red arrow), along with the longitudinal course of the LLC and its communication with the lingual cortical surface.

**Figure 6:**
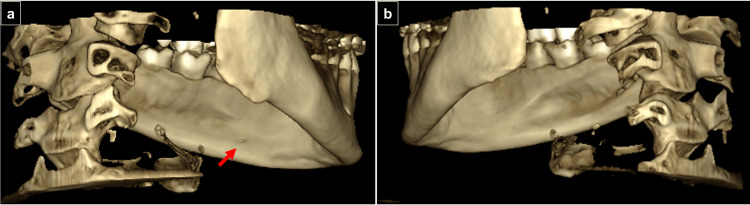
Cone-beam computed tomography (CBCT) imaging of the mandible. Three-dimensional reconstruction showing (a) External cortical opening of the lateral lingual foramen (LLF) only on the left side (red arrow); and (b) Absence of LLF on the right side.

During routine evaluation of the CBCT images, an incidental finding of LLF was identified in the left mandible. The foramen was located lingual to the mental foramen and apical to the premolar roots on the lingual cortical surface, suggesting the presence of a neurovascular structure branching from the mental or inferior alveolar neurovascular complex (Figure 2). Coronal sections demonstrated a distinct intrabony canal (LLC) traversing the lingual cortex medial to the IAN canal and mental foramen (Figure 3 and Figure 4). Axial and sagittal views confirmed continuity between this canal and the overlying lingual soft tissues, consistent with a true LLF and associated LLC (Figure 1, Figure 5, and Figure 6a). The dimensions of the LLF and LLC in this case were approximately 0.93 mm in diameter and 6.39 mm in length. Additionally, the vertical distance of the LLF to the inferior border of the mandible was 8.19 mm. CBCT evaluation revealed that the LLF and LLC were unilateral (left-sided) in the present case (Figures 6a, 6b).

**Figure 7:**
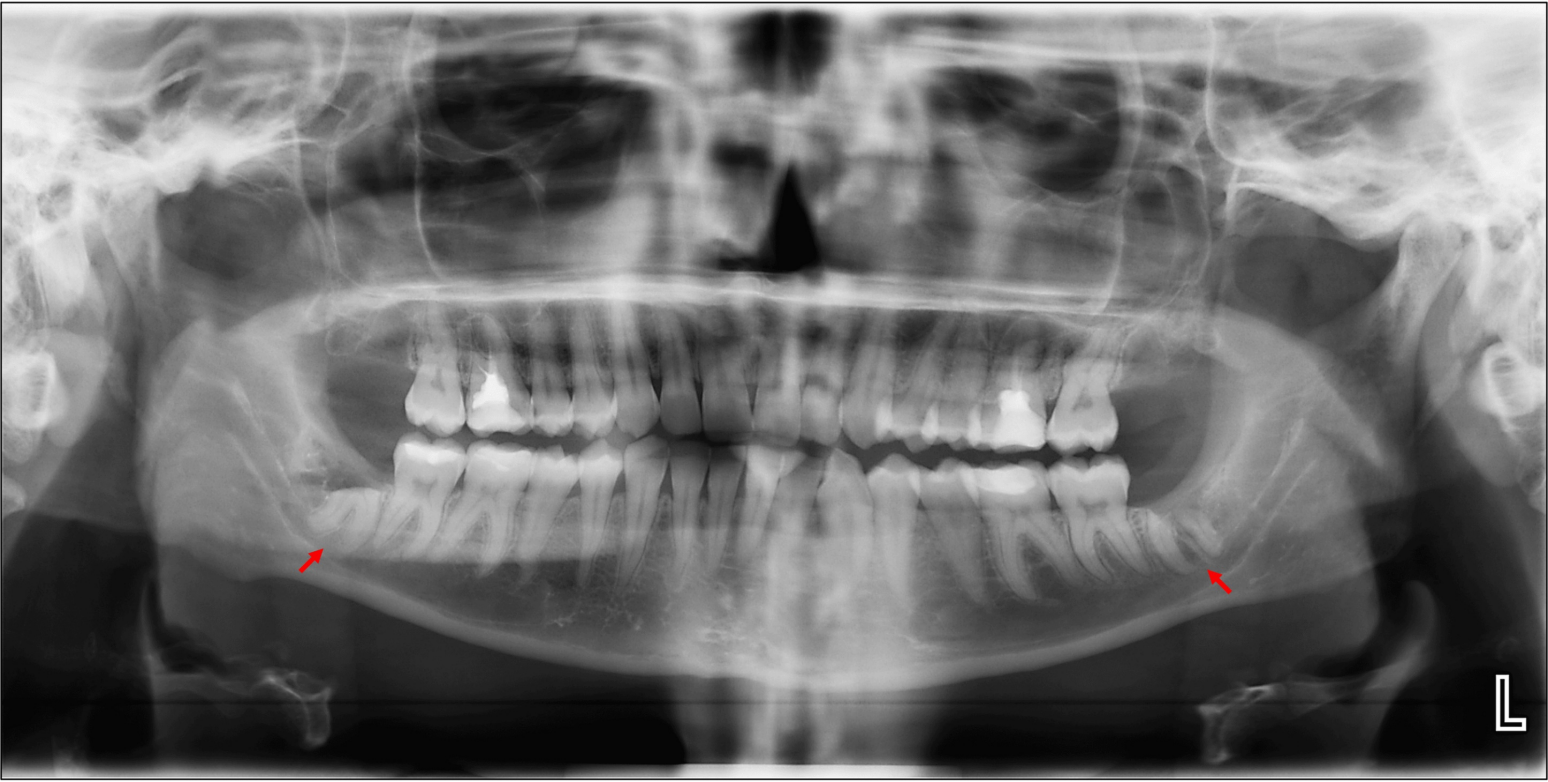
Dental panoramic radiograph (OPG) Postoperative orthopantomogram (OPG) showing bilateral mandibular third molar roots (red arrows), after coronectomy.

The patient subsequently underwent uncomplicated simple extractions of the maxillary third molars. Given the proximity of the mandibular third molar roots to the IAN canal, coronectomies were performed bilaterally under local anesthesia (Figure 7). The intraoperative and postoperative courses were uneventful, and the patient reported no neurosensory disturbances. Upon completion of care, the patient was referred back to her orthodontist with documentation highlighting the presence of the left LLF. Precautionary instructions, specifically regarding floor-of-mouth swelling, bleeding, and/or neurosensory deficits, were provided should future treatment planning involve procedures such as mandibular osteotomy, genioplasty, or implant placement in the interforaminal region.

## Discussion

A review of pertinent literature on LLF, its diagnosis, and clinical implications was conducted using a focused search of the PubMed database. PubMed was selected for its extensive collection of evidence-based reports and its ability to rapidly search the literature for pertinent reports on the topic. The search terms included “Lateral lingual foramen”, “Lateral lingual canal”, “Mandible”, “CBCT”, “Oral surgery”, and “Dental implantology”, along with different combinations of Boolean operators. The search filters included English-language articles published within the last 20 years (2006 - 2025), with no restrictions on the report type (reviews, original studies including clinical, cadaveric, and radiographic data, case reports, and case series). From the articles identified through preliminary searching, duplicates and studies deemed irrelevant based on abstract screening were excluded. With a primary focus on reviewing original studies and case series reporting on the incidence and clinical implications of LLF, 20 studies were selected for the literature review. The review findings presented in Table 1 provide a qualitative synthesis of published findings emphasizing anatomical patterns and clinical relevance rather than exhaustive methodological comparison.

**Table 1:** Summary of pertinent findings, clinical implications, and surgical risks associated with the lateral lingual foramen (LLF) and lateral lingual canal (LLC) Studies included in this table comprised radiographic, cadaveric, and clinical investigations using CT or CBCT imaging, with heterogeneous sample sizes and dentate status as reported by individual authors

Author (Year)	Pertinent Findings	Clinical Implications	Surgical Risks
Tagaya et al. (2009) [17]	LLF present in 80% of patients (n = 160/200); predominantly in the premolar region; often coinciding with the mental foramen.	CT/CBCT recommended to identify LLF pre-surgically.	Severe hemorrhage; risk of lingual perforation.
Nakajima et al. (2014) [12]	LLF supplied by submental/sublingual arterial branches.	Understanding vascular supply supports surgical planning.	Hemorrhage into sublingual/submandibular spaces, airway obstruction.
Yildirim et al. (2014) [7]	LLF present in 21.1% patients (n = 135/639); mean diameter 0.84 mm; 24% >1 mm; mean vertical distance from the LLF to the inferior border of the mandible - 17.4 mm.	3D imaging assesses bleeding risk; large diameters higher risk.	Hemorrhage; lingual perforation risk in atrophic mandibles.
Uchida et al. (2015) [10]	Alveolar crest-to-LLF distance in edentulous patients (mean - 15.7 mm) was significantly shorter than in dentulous mandibles (mean - 19.3 mm).	Anticipate bleeding in resorbed ridges.	Hemorrhage with airway compromise.
Wang et al. (2015) [8]	LLF present in 99% patients (n = 100/101); common in incisor–canine region; larger diameters in males.	Diameter knowledge improves osteotomy/implant safety.	Life-threatening bleeding; neurosensory issues.
He et al. (2016) [6]	LLF leading to LLC present in 14.9% patients (n = 102/685); most below tooth apices.	Assessment crucial due to neurovascular contents.	Hemorrhage, hematoma, mylohyoid nerve injury.
Sanomiya Ikuta et al. (2016) [18]	LLF present in 39% patients (n = 218/558); frequent at second premolar; mean vertical distance from the LLF to the inferior border of the mandible - 5.82 mm.	CBCT reliable for identifying variation.	Hemorrhage, hematoma, airway obstruction.
Iwanaga et al. (2018) [19]	LLF draining a large inferior alveolar vein from the bifid canal.	Rare variation requiring caution.	Severe bleeding due to large venous drainage.
Krishnan et al. (2018) [20]	LLF present in 20.2% patients (n = 22/109); predominantly apical to first premolar; LLC connected to the mandibular canal.	CBCT mapping required in the mental region.	Complications in premolar region surgery.
Moro et al. (2018) [16]	LLF leading to LLC present in 75.9% patients (n = 44/58); mostly in canine-premolar region; mean diameter of LLF - 0.81 mm.	Avoid injury to small neurovascular canals.	Sensory disturbances, bleeding.
Trost et al. (2020) [1]	LLF present in 38.9% patients (n = 179/460); the diameter of LLF and length of LLC were shorter than those of MLF and MLC, respectively; LLF of critical diameter (>1 mm) in 65%; 84% LLF below mental spine.	Higher risk in edentulous patients; 3D imaging advised.	Severe bleeding; airway obstruction.
Wei et al. (2020) [15]	LLF present in 69.9% patients (n = 314/306) and bilateral in 29.1%; mostly apical to premolars; mean diameter of LLF - 0.9 mm; mean vertical distance from the LLF to the inferior border of the mandible - 7.3 mm (male)/6.9 mm (female).	LLFs >1 mm require caution.	Paresthesia or hemorrhage.
Aytuğar & Keskek (2021) [9]	LLF present in 54.3% patients (n = 396/741) and bilateral in 38.9%; predominantly apical to premolars (87.6%) and more commonly apical to second premolar; mean vertical distance from LLF to inferior border of mandible - 4.71 mm.	Imaging reduces premolar complications.	Bleeding, hematoma, edema, airway risk.
Moshfeghi et al. (2021) [2]	LLF present in 11.5% patients (n = 23/200); commonly apical to second premolar; more prevalent in males.	Knowledge prevents excessive bleeding/nerve injury.	Bleeding, neurosensory risk in atrophic ridges.
Silvestri et al. (2022) [13]	LLF identified in 73 out of 100 CBCT scans of edentulous mandibles; LLF supplied by neurovascular bundle from IAN; 30% >1 mm.	Risk unchanged by edentulism; resorption increases vulnerability.	Neurosensory issues, hemorrhage.
Taschieri et al. (2022) [14]	LLF present in 37.3% patients (n = 112/300); more commonly in the premolar region.	Variation requires cautious planning.	Massive bleeding; life-threatening cases.
Padhye et al. (2023) [4]	LLF identified in 149 out of 400 CBCT scans of mandibles; more commonly in the canine-premolar region; mean diameter of LLF - 0.88 mm; mean length of LLC - 6.26 mm.	CBCT essential before anterior mandibular surgery.	Fatal bleeding risk due to sublingual space proximity.
Mostafavi et al. (2024) [3]	LLF prevalence was 5.91% (68 patients) out of 2082 CBCT scans assessed; more commonly in the first premolar region; mean diameter of LLF - 0.99 mm,	Identification reduces nerve injury risk.	Bleeding, paresthesia, hematoma.
Baghele et al. (2025) [11]	LLF identified in 18 out of 100 CBCT scans of mandibles; mostly in the canine–first premolar region; mean vertical distance from the LLF to the inferior border of the mandible - 7.1 mm.	CBCT reduces hemorrhage risk.	Bleeding into floor of mouth; airway obstruction.
Tsatsarelis et al. (2025) [6]	A total of 52 LLF identified in 96 dry mandibles; more commonly in the canine-premolar region; mostly small (about 0.4 mm) but clinically relevant; mean vertical distance from LLF to inferior border of mandible - 9.71 mm (right) / 10.21 mm (left).	Anatomy aids preoperative planning.	Hemorrhage, neurosensory issues; airway obstruction risk.
LLF: Lateral Lingual Foramen; LLC: Lateral Lingual Canal; MLF: Median lingual foramen; MLC: Median Lingual Canal; IAN: Inferior Alveolar Nerve; CBCT: Cone-Beam Computed Tomography; CT: Computed Tomography			

Prevalence, location, and morphological characteristics of the lateral lingual foramen

The anatomical variability of lingual foramina, particularly the LLF, has been extensively documented across multiple studies. A synthesis of the reviewed literature reveals distinct patterns in prevalence, spatial distribution, and morphological features, which are critical for both anatomical understanding and clinical applications (Table 1). Across the 20 reviewed studies, the LLF and LLC were consistently identified as frequently encountered, but highly variable anatomical structures on the lingual cortex of the mandible. Reported prevalence ranged widely (6-99%), reflecting differences in imaging modalities, populations, diagnostic criteria, and methodological heterogeneity. The aforementioned prevalence range is presented descriptively and should be interpreted in the context of heterogeneous study designs, sample sizes, and imaging protocols. It must further be noted that the differences in imaging modality, CBCT field of view, voxel size, diagnostic thresholds, study populations, and criteria used to distinguish true lingual foramina from nutrient canals contribute significantly to these discrepancies.

Anatomically, most LLFs were located in the premolar or canine regions, with diameters typically between 0.4 and 1.2 mm; however, a significant proportion (>1 mm) posed a higher risk for hemorrhagic complications. LLFs were predominantly unilateral, and bilateral in only about 29-39% of cases. The length of the LLC ranged around 6 mm, and both the diameter of LLF and the length of LLC were relatively less than that of the median lingual foramen (MLF) and canal (MLC). The LLF frequently transmitted branches of the submental or sublingual arteries, or in rare cases, large venous structures originating from bifid mandibular canals. Studies emphasized the importance of CBCT for reliable detection and preoperative mapping, noting greater vulnerability in edentulous mandibles due to reduced vertical bone height and the presence of superficial neurovascular structures. Based on radiographic assessment, while the distance of the LLF from the alveolar crest ranged from 15 to 19 mm, it was around 4.7 - 17 mm between the LLF and the inferior border of the mandible. Both the above measurements were relatively lower in female patients, owing to the small overall anatomical dimensions of the female mandible. Comparing dentate and edentulous mandibles, alveolar bone resorption secondary to edentulism remarkably reduced the distance between the alveolar crest and LLF (Table 1).

Clinically, the LLF/LLC complex is implicated in potentially life-threatening complications during implant placement, osteotomy, genioplasty, and other oral surgical procedures, particularly due to the risk of rapid floor-of-mouth hemorrhage and airway compromise [6]. Collectively, the literature underscores the need for routine preoperative assessment for an incidental finding of LLF and LLC to prevent bleeding, neurosensory deficits, and procedural complications [14]. Morphologically, LLFs are associated with bony canals that transmit neurovascular bundles. These canals exhibit diverse trajectories, with some coursing horizontally toward the incisive region and others ascending vertically to anastomose with branches of the sublingual artery [9]. The diameter and length of these canals also vary, with lateral canals generally being shorter and narrower than midline canals (Table 1). Such morphological distinctions may influence the hemodynamic significance of the LLF, as smaller canals might harbor less prominent vasculature, though this hypothesis requires further validation [15].

CBCT-based assessment of lateral lingual foramina and related structures

The reviewed studies collectively affirm CBCT as the gold standard for assessing lingual foramina, particularly for its ability to visualize small or obliquely oriented canals that may harbor clinically significant vasculature [15]. CBCT has emerged as a pivotal tool for evaluating the LLF and its associated anatomical structures, offering superior spatial resolution and three-dimensional visualization compared to conventional radiography [17]. CBCT is not only helpful in identifying LLF but is also useful for characterizing LLC and mapping its neurovascular relationships, which are critical for preoperative planning and risk mitigation in oral surgical procedures (Table 1). CBCT imaging is further reported to play a crucial and supporting role in craniofacial volumetric assessment and standardized imaging workflows for surgical planning [21].

One study specifically employed CBCT to assess the localization and prevalence of the LLF, reporting its consistent identification along the lingual cortical plate with distinct canal trajectories [9]. The high-resolution capabilities of CBCT enabled precise measurements of canal dimensions and orientations, revealing that LLCs often exhibit horizontal or oblique courses, in contrast to the vertical pathways of midline canals. Another investigation focused on the Indian population and used CBCT to document anatomical variations in lingual foramina and their bony canals, noting that the vast majority of cases presented with two foramina, underscoring the importance of CBCT in detecting accessory structures [4]. Studies based on CBCT have also proposed a critical diameter threshold of greater than 1 mm for the LLF or LLC, beyond which the risk of clinically significant hemorrhage increases due to the likelihood of larger neurovascular bundles traversing the canal [6,7,15]. Accurate identification of such high-risk foramina depends heavily on CBCT acquisition parameters [4]. Compared to two-dimensional radiography, CBCT allows precise assessment of canal morphology, cortical perforation, and spatial relationships to adjacent neurovascular structures, thereby facilitating improved preoperative risk stratification and surgical planning [20].

The utility of CBCT extends beyond mere detection; it facilitates the differentiation of the LLF from other mandibular foramina, such as the mandibular incisive canal. For instance, a study employed CBCT to delineate the mandibular incisive canal and its spatial relationship to the lingual foramen, highlighting the potential for neurovascular conflict during implant placement [9]. Studies have also measured distances between the canal and critical landmarks (e.g., the lingual plate and inferior mandibular border), demonstrating CBCT’s role in quantifying anatomical relationships that are invisible to two-dimensional imaging [1-4,8,13,14,18]. The integration of CBCT into clinical practice is further supported by its ability to reduce procedural complications. For example, the detection of LLF and LLC with CBCT can alert surgeons to potential bleeding risks, prompting modifications in osteotomy or implant placement techniques [4]. Nevertheless, standardized protocols for CBCT acquisition and interpretation remain lacking, necessitating future research to optimize imaging parameters and diagnostic criteria for the LLF and its variants [11].

Clinical implications of LLF in oral surgical and implantological procedures

The LLF and its associated neurovascular structures pose significant considerations for oral surgical interventions, particularly in implantology and osteotomy procedures. The reviewed literature highlights the potential complications arising from inadvertent damage to these anatomical features, emphasizing the need for preoperative assessment and tailored surgical planning (Table 1).

One critical clinical implication involves the risk of hemorrhage during surgical procedures involving the anterior mandible. The LLF often transmits branches of the sublingual or submental arteries, which, if severed, can lead to significant bleeding [12]. A study by Moro et al. demonstrated that the LLC frequently harbors sizable vascular bundles greater than a critical diameter of 1 mm [16]. This finding underscores the importance of identifying these structures before procedures such as genioplasty or anterior mandibular osteotomies, where blind instrumentation may compromise the vascular supply [12,16,17]. Moreover, the study reported that accessory foramina, when present, may contain additional neurovascular elements, further increasing the complexity of surgical interventions [16].

Beyond hemorrhage, sensory disturbances represent another notable concern. Although the LLF is primarily associated with vascular structures, some studies suggest that it may also transmit sensory nerve fibers originating from the lingual or mylohyoid nerves [15]. Damage to these fibers during implant placement or bone grafting procedures could result in transient or permanent paresthesia in the anterior mandibular region [9]. This risk is exacerbated when the LLF exhibits an aberrant course or multiple branching patterns, as these variations are often undetectable on conventional radiography [14,15,17,20]. The integration of advanced imaging, particularly CBCT, into clinical workflows has proven instrumental in mitigating these risks. For instance, in cases where the LLF is positioned unusually close to the alveolar crest, altering the implant angulation or opting for shorter fixtures may prevent inadvertent perforation [4,5,10]. Future investigations should aim to correlate anatomical variations of the LLF with clinical outcomes, thereby refining risk stratification and enhancing procedural safety for minimizing complications in oral surgical and implantological practice [4,7].

Recommendations and limitations

The collective evidence suggests that the LLF is not merely an anatomical curiosity but a clinically relevant structure whose unpredictable nature warrants careful preoperative assessment [6]. The implications of these findings are both theoretical and practical. From a theoretical standpoint, the high degree of variability in LLF anatomy challenges conventional models of mandibular neurovascular distribution [9]. This has ramifications for understanding hemodynamic pathways in the anterior mandible, particularly in scenarios involving trauma or surgical manipulation. In practice, the findings underscore the need to incorporate advanced imaging, specifically CBCT, into routine preoperative planning for procedures such as implant placement or osteotomies [4,9].

Nevertheless, several limitations in the current literature must be acknowledged. The predominance of cross-sectional and cadaveric studies in this review introduces potential biases related to sample selection and imaging protocols. For instance, studies relying on CBCT may overestimate the prevalence of smaller foramina because of its higher resolution. In contrast, cadaveric dissections might miss subtle vascular variations that are only apparent in living tissue. These methodological constraints highlight the need for standardized diagnostic criteria to validate the observed patterns across diverse populations. Yet another limitation is the heterogeneity in study design, imaging modality, CBCT acquisition parameters, diagnostic thresholds, and population characteristics. Therefore, the reported prevalence and morphometric values across studies should be interpreted descriptively, and any comparative estimation should be done with caution.

From a future perspective, there is a primary need for large multicentric case series and longitudinal studies correlating LLF anatomy with clinical outcomes, such as postoperative bleeding rates or sensory complications. Such investigations would provide empirical evidence to guide risk stratification and surgical decision-making. Secondly, ultrasound or angiographic studies are required to understand the hemodynamic significance of the LLF and LLC. Finally, the development of AI-assisted CBCT analysis tools could enhance the detection and classification of lingual foramina, reducing inter-observer variability and improving diagnostic accuracy.

## Conclusions

This case highlights the clinical importance of identifying the LLF and its associated canal (LLC) as an incidental but potentially important anatomical variation during CBCT evaluation of the mandible. Although rarely encountered, the LLF is a neurovascular structure that can cause significant intraoperative bleeding or neurosensory complications if unrecognized during procedures such as implant placement, genioplasty, or oral surgeries around the mental foramen. This is particularly possible in the presence of larger-diameter canals, reduced mandibular bone height, or atrophic edentulous ridges. The reviewed literature demonstrates considerable variability in LLF prevalence and morphology, underscoring the importance of individualized risk assessment rather than assuming uniform surgical risk. CBCT facilitates reliable identification and characterization of the LLF and should be considered during preoperative planning for anterior mandibular surgery and implant placement to enhance surgical predictability and minimize avoidable complications.
